# CT-Derived Paraspinal Muscle Asymmetry Is Associated with Deformity Severity in Adolescent Idiopathic Scoliosis: A Quantitative CT Study

**DOI:** 10.3390/jcm15083084

**Published:** 2026-04-17

**Authors:** Chong Zhao, Zhenqi Zhu, Haiying Liu, Shuai Xu

**Affiliations:** 1 Department of Spinal Surgery, Peking University People’s Hospital, Peking University, Beijing 100871, China; 2 Department of Spinal Surgery, Peking University International Hospital, Beijing 100084, China

**Keywords:** adolescent idiopathic scoliosis, paraspinal muscles, Cobb angle, vertebral rotation, muscle asymmetry

## Abstract

**Objectives:** To characterize paraspinal muscle asymmetry using quantitative CT parameters in adolescent idiopathic scoliosis (AIS) and to examine the associations among muscle asymmetry, vertebral rotation, and curve severity. **Methods:** A retrospective analysis of 68 AIS patients was conducted. Quantitative CT measured the fatty infiltration rate (FIR) of paraspinal muscles at apical and stable vertebral levels. A muscle asymmetry index was calculated based on the FIR difference between concave and convex sides. Pearson and Spearman correlations and linear regression were used as the main analyses. AVR across upper, pedicle, and lower levels was evaluated using repeated-measures analysis, and SEM was performed as an additional analysis. Inter-observer repeatability of the apical muscle measurements was additionally assessed using an independently repeated segmentation dataset. **Results:** Paraspinal muscles at the apical region exhibited significant asymmetry, with concave FIR (33.4 ± 14.0%) being significantly higher than convex FIR (15.8 ± 6.8%; *p* < 0.001). In contrast, the stable vertebra showed no significant asymmetry. Muscle asymmetry was significantly associated with Cobb angle in both Pearson and Spearman analyses (r = 0.456, *p* = 0.0001; rho = 0.430, *p* = 0.0003). Its association with AVR was weaker (Pearson r = 0.232, *p* = 0.058; Spearman rho = 0.302, *p* = 0.013). In multivariable linear regression, both AVR (β = 1.222, 95% CI 0.827 to 1.617, *p* < 0.001) and muscle asymmetry (β = 0.375, 95% CI 0.167 to 0.583, *p* = 0.0006) remained independently associated with Cobb angle. Inter-observer repeatability for the apical muscle asymmetry index remained excellent (ICC(2,1) = 0.958, 95% CI 0.933 to 0.974). **Conclusions:** Significant asymmetric CT-derived low-attenuation change was observed in the apical paraspinal muscles of patients with AIS, predominating on the concave side. In this severe AIS cohort, paraspinal muscle asymmetry was consistently associated with Cobb angle and showed a weaker, more method-dependent association with AVR. These findings suggest a relationship between paraspinal muscle asymmetry and the severity of three-dimensional deformity.

## 1. Introduction

Adolescent idiopathic scoliosis (AIS) is the most prevalent spinal deformity during adolescence, affecting approximately 2–3% of the global population [1]. AIS is not simply a coronal deviation but a complex three-dimensional deformity involving multiple planes. It is characterized not only by lateral curvature but also by abnormalities in the sagittal physiological profile and significant vertebral axial rotation (AVR) [2,3]. In accordance with the Hueter-Volkmann law, asymmetric mechanical loading modulates skeletal growth, leading to vertebral wedging and abnormal distribution of local bone mineral density. This finding indicates that vertebral rotation and the subsequent modification of the mechanical environment are not merely sequelae of the deformity, but also closely linked to deformity severity [4,5]. Consequently, vertebral rotation is regarded as the core component of the three-dimensional structural anomaly in AIS, being closely associated with cosmetic deformity, cardiopulmonary compromise, and the complexity of treatment [6].

Early studies on AIS focused largely on skeletal anomalies, including genetics, hormonal regulation, and spinal growth modulation [7,8]. In this context, the observed decrease in paraspinal muscle volume, morphological alterations, and functional asymmetry have traditionally been interpreted as secondary adaptations to the established spinal deformity—specifically, atrophy due to compression on the concave side and compensatory stretching on the convex side [9,10]. Nevertheless, the purely “secondary adaptation” hypothesis has limitations. Recent physiological evidence indicates that patients with AIS exhibit delayed paraspinal muscle activation during rapid movements [11], accompanied by prolonged oxygenation recovery times and a loss of slow-twitch muscle fibers on the concave side, thereby suggesting underlying metabolic dysfunction [12,13,14]. These functional impairments may accompany or reflect severe bony deformity and cannot be fully accounted for by secondary adaptive changes alone. A body of research has previously been conducted on bone mineral density and its relationship to systemic metabolic abnormalities. Patients with AIS have been shown to exhibit distinct segmental variations in bone loss at the apical region. This suggests that the local mechanical environment plays a pivotal role in tissue remodeling [5,15]. In light of this, it has been hypothesized that paraspinal muscles may exhibit similar segmental pathological characteristics that warrant quantitative evaluation.

These findings have drawn attention to the role of paraspinal muscles in AIS pathogenesis. As critical dynamic stabilizers of the spine, the paraspinal muscles, particularly the deep groups such as the multifidus and rotatores, are essential for maintaining three-dimensional vertebral alignment and providing rotatory stability [6]. Asymmetric CT-derived muscle composition changes of the paraspinal musculature at the apical level may related to deformity severity and local mechanical imbalance [11,16,17]. However, because CT-derived composition metrics are surrogate measures rather than direct histologic or MRI validation, and because the present study is cross-sectional, these relationships should be interpreted as associations rather than evidence of temporality.

Although previous cross-sectional studies have indicated a significant correlation between the degree of paraspinal muscle composition asymmetry and both curve severity and vertebral rotation [1,18], there remains a paucity of research utilizing high-precision quantitative imaging to systematically evaluate how muscle asymmetry is linked to three-dimensional deformity formation. Therefore, this study aims to employ quantitative CT analysis to precisely characterize paraspinal attenuation-based surrogates of muscle composition at both the apical and stable vertebral levels in patients with AIS, utilizing the stable vertebra as an internal control. Furthermore, we examined the relationships among muscle asymmetry, vertebral rotation, and curve severity using correlation and linear regression analyses, and performed SEM as an additional analysis. Our objective is to provide imaging evidence for interpretation of associations of the muscle-bone relationship in severe AIS.

## 2. Methods

### 2.1. Study Population

This retrospective study was approved by the Ethics Committee of Peking University People’s Hospital (Approval No. 2024PHB103-001). We reviewed the records of patients diagnosed with AIS at our institution between January 2018 and December 2023. The inclusion and exclusion criteria were as follows:

Inclusion criteria: (1) Confirmed diagnosis of AIS with a major curve located in the thoracic or thoracolumbar region; (2) Availability of complete imaging data, including standing full-spine radiographs and whole-spine CT scans; (3) No history of prior spinal surgery.

Exclusion criteria: (1) Non-idiopathic scoliosis (e.g., congenital, neuromuscular, or syndromic etiology); (2) History of spinal trauma, infection, or spinal tumor; (3) Presence of significant artifacts on CT images that interfered with muscle boundary identification or density measurement.

A total of 68 patients with complete muscle asymmetry, AVR, and Cobb angle data were included in the analysis.

### 2.2. Imaging Assessment

To systematically evaluate the overall severity of the spinal deformity and its axial rotation characteristics, a multidimensional imaging assessment combining standing radiographs and CT imaging was performed. Standard standing full-spine posteroanterior and lateral radiographs were obtained for all patients to measure the main curve Cobb angle for the assessment of overall deformity severity. Patients underwent spinal helical CT scanning in the supine position. The examination was performed using a GE Discovery three-dimensional reconstructive CT scanner (GE Healthcare, Chicago, IL, USA), with a tube voltage of 120 kV, a slice thickness of 1.25 mm, and a spacing of 0.625 mm. All image data were exported in DICOM format for subsequent analysis.

**Figure 1:**
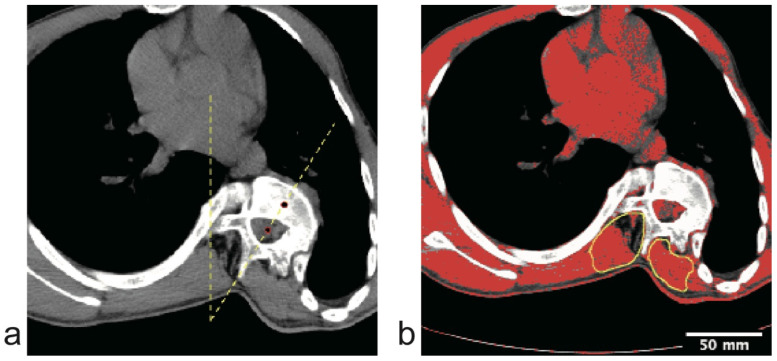
Illustration of quantitative CT measurement methods. (**a**) Measurement of Axial Vertebral Rotation (AVR) using the modified Aaro-Dahlborn method. The angle is defined between the sagittal midline (vertical dashed line) and the axis connecting the geometric center of the vertebral body and the spinal canal (red dots). (**b**) Segmentation of paraspinal muscles. The region of interest (ROI, yellow outline) includes the multifidus and erector spinae. Tissue within the ROI was segmented using a dual-threshold method to derive the CT low-attenuation proportion used in this study.

AVR was measured on axial CT images using the classic modified Aaro-Dahlborn method (Figure 1a). The specific procedure involved identifying the vertebral body-spinal canal axis, defined as the line connecting two points: (A) the posterior point, representing the geometric center of the spinal canal; and (B) the anterior point, representing the geometric center of the vertebral body. AVR was defined as the angle between this axis and the sagittal midline. To verify the segmental specificity of the deformity, AVR was measured separately for the apical, upper end, and lower end vertebrae. Comparisons across the upper, pedicle, and lower levels were analyzed using repeated-measures statistics.

### 2.3. CT-Based Quantitative Analysis of Paraspinal Muscles

Given the crucial role of paraspinal muscles in maintaining spinal stability and modulating three-dimensional deformity in AIS, this study performed a quantitative analysis of the morphological and attenuation-based composition characteristics of both concave and convex paraspinal muscles based on CT imaging. All image analyses were performed using Fiji (version 20260307-1417, ImageJ, National Institutes of Health, Bethesda, MD, USA). The regions of interest (ROIs) were manually outlined along the fascial boundary of the multifidus and erector spinae muscles (Figure 1b).

#### 2.3.1. Selection of Measurement Levels

To minimize the impact of anatomical variations or positional factors associated with single-slice measurements and to enhance the stability and representativeness of the data, three consecutive axial levels were selected for each target vertebra (the apical and stable vertebrae). The mean value of these measurements was used for analysis. The selected levels were: (1) the mid-pedicle level; (2) the level of the superior intervertebral foramen; and (3) the level of the inferior intervertebral foramen.

#### 2.3.2. Tissue Segmentation and Parameter Calculation

To ensure measurement reproducibility, all images were standardized to a soft tissue window setting with a window level of 40 Hounsfield units (HU) and a window width of 400 HU. Under these conditions, the ROI was manually outlined along the muscle fascial boundary by the same experienced orthopedic surgeon. A dual-threshold segmentation method was subsequently employed to distinguish tissue components, where the CT attenuation range for adipose tissue was defined as −110 HU to −35 HU based on preliminary experiments and anatomical references. Pixels falling within this range were interpreted as a low-attenuation fat-related component, while those exceeding this range were classified as functional muscle cross-sectional area. Accordingly, the Total Muscle CSA was defined as the aggregate area of all soft tissues within the ROI. Finally, the FIR abbreviation was retained for continuity, but it was used as a CT-derived low-attenuation proportion quantified using the formula: (Total Muscle CSA − Functional Muscle CSA)/Total Muscle CSA × 100%. We additionally performed a supplementary threshold-sensitivity analysis in 30 patients by repeating FIR derivation with an alternative adipose attenuation range of −120 to −30 HU and comparing the resulting FIR values and patient-level asymmetry estimates with those from the primary −110 to −35 HU definition.

#### 2.3.3. Muscle Asymmetry Index

To directly reflect the absolute difference in the low-attenuation proportion between the concave and convex sides at the same level, and to avoid the amplification effect inherent to ratio-based indices in cases of low baseline values, the Muscle Asymmetry Index was constructed using the difference in FIR. The calculation is as follows: Asymmetry Index = FIR_Concave − FIR_Convex. This index is expressed in percentage points. A positive value indicates a higher degree of low-attenuation change on the concave side compared to the convex side. The final data included in the statistical analysis represented the mean of the asymmetry indices from the three measurement levels (the core level and the superior/inferior auxiliary levels) at the apical region.

#### 2.3.4. Assessment of Measurement Reliability

To ensure the accuracy and reproducibility of the measurements, all imaging parameters (including Cobb angle, AVR, and muscle morphological parameters) were measured independently in a blinded fashion by two spinal surgeons, each with over 5 years of experience. The mean of the two measurements was used for the final analysis. In cases of significant discrepancy (inter-observer error > 5%), the data were reviewed and adjudicated by a senior chief surgeon to determine the final value. Inter-observer repeatability was additionally evaluated in an independently repeated apical muscle segmentation dataset derived from the same imaging workflow but re-measured in a blinded fashion by a second observer. The primary repeatability endpoint was the derived patient-level apical muscle asymmetry index, whereas raw ROI area was analyzed as a supplementary segmentation check.

### 2.4. Statistical Analysis

All statistical analyses were performed using R software (version 4.4.2). Continuous variables were presented as mean ± standard deviation (SD). The normality of data distribution was first assessed using the Kolmogorov–Smirnov test. To compare differences in morphology and CT-derived low-attenuation proportion characteristics between concave and convex paraspinal muscles, paired *t*-tests were used for normally distributed variables (such as muscle CSA), whereas the Wilcoxon signed-rank test was employed for non-normally distributed variables (such as FIR). Pearson and Spearman correlation coefficients were calculated to evaluate the relationships among paraspinal muscle asymmetry, AVR, and Cobb angle.

Inter-observer repeatability for the primary apical muscle asymmetry index and the supplementary raw ROI area measurements was evaluated using intraclass correlation coefficients.

Linear regression models were then used to evaluate the associations of muscle asymmetry with AVR and Cobb angle. Model diagnostics included residual normality, influential observations, and variance inflation factors. Repeated-measures ANOVA with Greenhouse-Geisser correction was used for AVR comparisons across the upper, pedicle, and lower levels. SEM with bootstrap confidence intervals was performed as an additional analysis. For the threshold-sensitivity analysis, we summarized FIR values under both thresholds, compared paired concave-convex differences, quantified patient-level agreement of the asymmetry index between thresholds, and examined whether correlation coefficients and linear-model estimates changed materially under the alternative threshold.

All statistical tests were two-tailed, and a *p*-value < 0.05 was considered statistically significant.

## 3. Results

### 3.1. Demographic and Baseline Characteristics

A total of 68 patients with AIS were included in this study. The baseline demographic characteristics and major radiographic parameters of the study population are summarized in Table 1. The mean age of the patients was 14.2 ± 3.1 years, and the mean body mass index (BMI) was 17.9 ± 2.7 kg/m^2^, reflecting the lean body habitus characteristic of the AIS population. Regarding deformity severity, the mean main curve Cobb angle was 76.6 ± 17.8°, and the mean AVR was 24.7 ± 8.1°, indicating that the cohort primarily consisted of moderate to severe scoliosis cases. Quantitative CT-based analysis of the paraspinal muscles revealed varying degrees of bilateral asymmetry in the apical region, with a mean muscle asymmetry index of 17.6 ± 15.5%.

### 3.2. Segmental Characteristics of Deformity and Muscle Composition Asymmetry at the Apical Region

**Figure 2:**
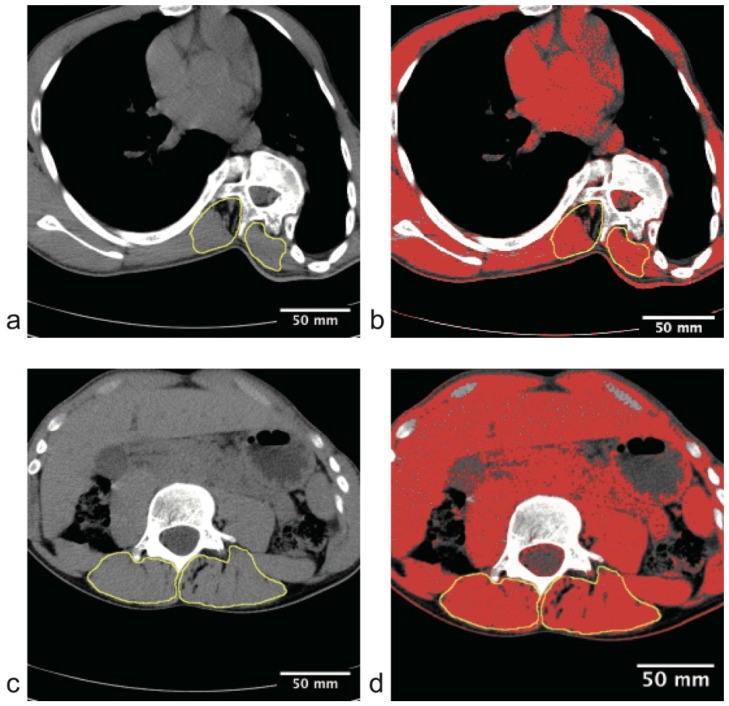
Representative CT images demonstrating segment-specific paraspinal muscle asymmetry in a patient with AIS. The paraspinal muscle areas are outlined by yellow circles. (**a**,**b**) Apical vertebral level. (**b**) Unenhanced axial CT image showing significant vertebral rotation. Note the visible hypodensity (darker areas) in the paraspinal muscles on the concave side (right), indicating greater low-attenuation signal. (**b**) Pseudo-color overlay where red pixels represent adipose tissue (−110 to −35 HU). The concave side exhibits extensive low-attenuation change compared to the convex side. (**c**,**d**) Stable vertebral level. (**c**) Axial CT image of the stable vertebra in the same patient, showing no rotation. (**d**) The pseudo-color map reveals symmetric muscle quality with minimal side-to-side difference (red pixels) on both sides, serving as an internal control. Note: This visually confirms that muscle asymmetry is localized to the deformity apex.

We first analyzed the distribution characteristics of spinal deformity and paraspinal muscle composition asymmetry across different anatomical segments. Representative CT images demonstrating these segmental characteristics are presented in Figure 2. Visually, the apical vertebra exhibits significant rotation accompanied by extensive low-attenuation change on the concave side (Figure 2a,b), standing in sharp contrast to the symmetric muscle quality observed at the stable vertebral level (Figure 2c,d).

**Figure 3:**
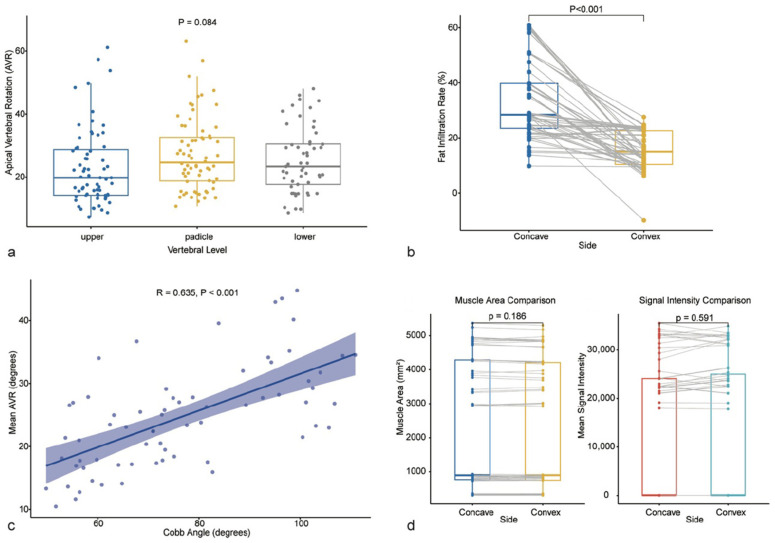
Quantitative analysis of segmental skeletal deformity and paraspinal muscle asymmetry. (**a**) Distribution of AVR across different vertebral levels. There was a trend toward higher AVR at the pedicle/apical level, but repeated-measures comparisons did not remain statistically significant after correction. (**b**) Comparison of FIR between concave and convex paraspinal muscles at the apical level. The concave side shows significantly higher low-attenuation proportion (*p* < 0.001, Wilcoxon signed-rank test). (**c**) Scatter plot showing a significant positive correlation between AVR and the main curve Cobb angle (r = 0.635, *p* < 0.001). (**d**) Comparison of paraspinal muscle characteristics at the stable vertebral level. No statistically significant differences were observed in muscle parameters between the concave and convex sides (*p* > 0.05, paired *t*-test), confirming that the asymmetric degeneration is localized to the apical region.

Consistent with this visual evidence, quantitative analysis of the entire cohort confirmed these segment-specific patterns. At the skeletal level, AVR demonstrated a distinct segment-specific distribution pattern. AVR across the upper, pedicle, and lower levels showed a trend toward higher rotation at the pedicle/apical level, but the overall repeated-measures comparison did not remain statistically significant after correction (overall *p* = 0.076; Greenhouse-Geisser corrected *p* = 0.088). At the muscular level, paraspinal muscle low-attenuation proportion in the apical region exhibited significant asymmetry (Figure 3b). Quantitative analysis revealed that the FIR of the concave paraspinal muscles was 33.4 ± 14.0%, significantly higher than the 15.8 ± 6.8% observed on the convex side (Table 2; Wilcoxon signed-rank test, *p* < 0.001). Furthermore, correlation analysis demonstrated a significant positive correlation between AVR and the main curve Cobb angle (r = 0.635, *p* < 0.001, Figure 3c), suggesting strong concordance between the degree of axial rotation and the severity of coronal curvature at the global level.

### 3.3. Verification of Muscle Pathology Specificity: Stable Vertebra Internal Control Analysis

To determine whether the observed paraspinal muscle asymmetry represents a local pathological change associated with the deformity rather than generalized muscle composition asymmetry across the entire spine, we performed an internal control analysis comparing the apical vertebra with the stable vertebra, which is removed from the center of the deformity. The results showed that at the stable vertebral level, the paraspinal muscles maintained good bilateral symmetry (Figure 3d). As detailed in Table 2, there were no statistically significant differences between the concave and convex sides of the stable vertebra regarding muscle CSA (2329 ± 1881 vs. 2320 ± 1867 mm^2^; *p* = 0.168) or mean signal intensity (13,055 ± 13,853 vs. 13,313 ± 14,055; *p* = 0.103). The stable-vertebra FIR surrogate also did not differ significantly between sides (33.0 ± 13.1% vs. 33.1 ± 13.1%; *p* = 0.551). These findings suggest that asymmetric paraspinal muscle composition asymmetry is predominantly localized to the apical region of the deformity, exhibiting clear segmental specificity.

### 3.4. Correlation Between Muscle Asymmetry and Skeletal Deformity Severity

**Figure 4:**
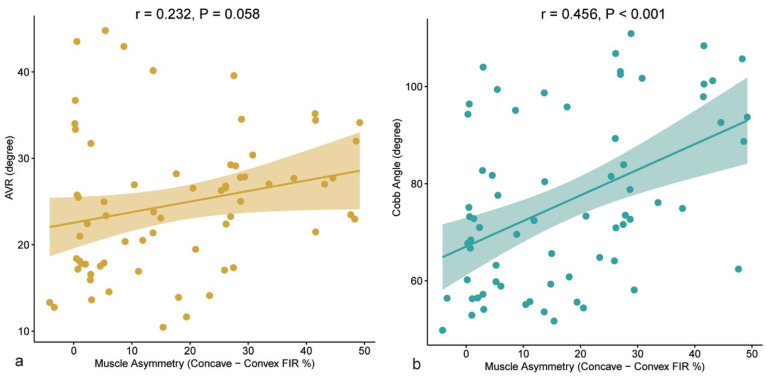
Correlations between paraspinal muscle asymmetry and skeletal deformity severity. (**a**) Scatter plot illustrating the relationship between the Muscle Asymmetry Index and AVR. A positive correlation was observed (r = 0.232, *p* = 0.058). (**b**) Scatter plot illustrating the relationship between the Muscle Asymmetry Index and the main curve Cobb angle. A significant positive correlation indicates that greater muscle imbalance is associated with more severe global spinal curvature (r = 0.456, *p* < 0.001). Pearson correlation coefficients and 95% confidence intervals (shaded areas) are displayed.

We further analyzed the relationship between the degree of paraspinal muscle asymmetry and the severity of skeletal deformity. Pearson correlation analysis indicated a positive trend between the muscle asymmetry index in the apical region and AVR, although this did not reach statistical significance (r = 0.232, *p* = 0.058, Figure 4a). Conversely, a significant positive correlation was observed between the muscle asymmetry index and the main curve Cobb angle (r = 0.456, *p* < 0.001, Figure 4b), suggesting that a higher degree of bilateral paraspinal muscle imbalance is generally associated with greater overall severity of spinal curvature. Spearman correlation analysis showed a similar association between muscle asymmetry and Cobb angle (rho = 0.430, *p* = 0.0003) and a modest association between muscle asymmetry and AVR (rho = 0.302, *p* = 0.013) (Table 3).

### 3.5. Linear Regression Models of Deformity Severity

To complement the correlation analyses, we fit three linear regression models, and the results are summarized in Table 4. In Model A (AVR ~ Muscle_Asymmetry), the association between muscle asymmetry and AVR was borderline (β = 0.122, 95% CI −0.005 to 0.249, *p* = 0.0584; R^2^ = 0.054). In Model B (Cobb ~ Muscle_Asymmetry), muscle asymmetry was significantly associated with Cobb angle (β = 0.524, 95% CI 0.271 to 0.778, *p* = 0.0001; R^2^ = 0.208). In Model C (Cobb ~ AVR + Muscle_Asymmetry), both AVR (β = 1.222, 95% CI 0.827 to 1.617, *p* < 0.001) and muscle asymmetry (β = 0.375, 95% CI 0.167 to 0.583, *p* = 0.0006) remained independently associated with Cobb angle, and the model explained 50.4% of the variance in Cobb angle. Model diagnostics showed that Model A had the weakest residual behavior, whereas Model C had the best residual behavior. No meaningful multicollinearity was observed in Model C (VIF = 1.057 for both predictors).

### 3.6. Inter-Observer Repeatability of Muscle Measurements

**Figure 5:**
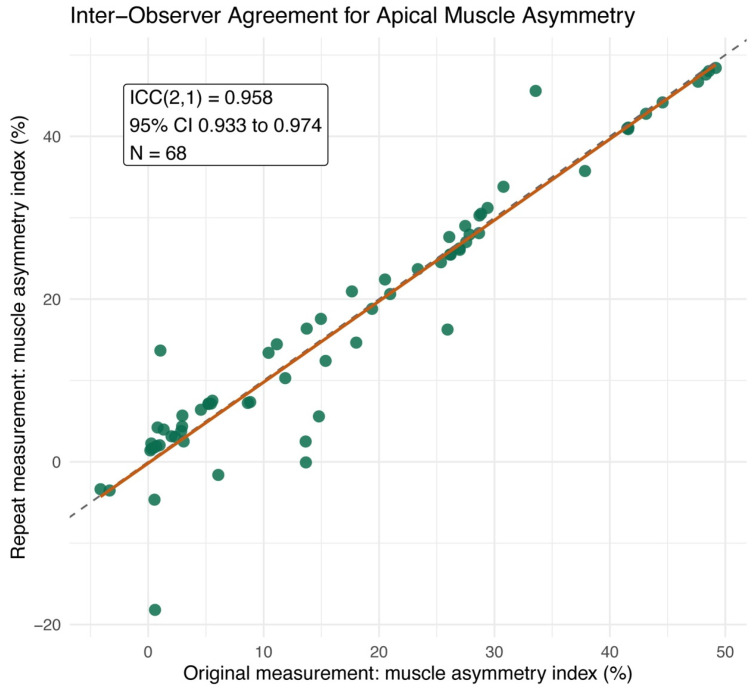
Inter-observer agreement for the primary apical muscle asymmetry endpoint. Scatter plot comparing the original and repeated apical muscle asymmetry measurements. The dashed diagonal line indicates identity. The primary repeatability analysis showed excellent agreement, with ICC(2,1) = 0.9579 (95% CI 0.9327 to 0.9738). Raw ROI area repeatability is reported separately in Table 5 as a supplementary segmentation check.

Inter-observer repeatability was excellent in 68 paired apical muscle measurement sets (Figure 5). For the primary patient-level apical muscle asymmetry index, ICCs indicated excellent agreement, and the supplementary raw ROI area measurements showed similarly high repeatability, supporting the robustness of the manually segmented apical muscle measurements used in this study.

**Table 5:** Inter-observer repeatability of apical muscle measurements.

Metric	Paired Measurements	ICC	95% CI
Primary endpoint: apical muscle asymmetry index	68	0.9579	0.9327 to 0.9738
Primary endpoint: apical muscle asymmetry index	68	0.9785	0.9652 to 0.9867
Supplementary check: raw ROI area	272	0.9996	0.9995 to 0.9997
Supplementary check: raw ROI area	272	0.9998	0.9998 to 0.9999

ICC: Intraclass correlation coefficient.

### 3.7. HU-Threshold Sensitivity Analysis

To quantify the effect of HU-threshold variation on the derived CT muscle metric, we performed a supplementary threshold-sensitivity analysis in 30 patients with paired apical and stable measurements (Supplementary Tables S1–S4). As expected, the stricter alternative threshold (−120 to −30 HU) yielded lower absolute FIR values than the primary definition (−110 to −35 HU) at both the apical level (concave 19.7% vs. 24.4%; convex 19.1% vs. 23.6%) and the stable level (concave 14.4% vs. 18.5%; convex 14.1% vs. 17.8%). However, agreement of the patient-level asymmetry index between thresholds remained high, with Pearson r = 0.959 and a mean absolute difference of 2.16 percentage points at the apex, and Pearson r = 0.965 with a mean absolute difference of 1.75 percentage points at the stable vertebra. The paired side-to-side comparisons remained non-significant under both thresholds (apex: *p* = 0.607 vs. *p* = 0.902; stable: *p* = 0.853 vs. *p* = 0.805). Downstream association estimates also changed little across thresholds: for apical asymmetry, the correlation with Cobb angle was −0.063 versus −0.042 and the correlation with mean AVR was −0.058 versus 0.004; in the joint model, the asymmetry coefficient remained non-significant (−0.032, *p* = 0.886 vs. −0.080, *p* = 0.700), whereas the AVR coefficient and overall model fit were essentially unchanged (β = 1.664 vs. 1.666; R^2^ = 0.637 vs. 0.639). These findings suggest that threshold variation shifted the absolute FIR level more than the relative patient-level asymmetry ranking in this supplementary analysis.

### 3.8. SEM Analysis

**Figure 6:**
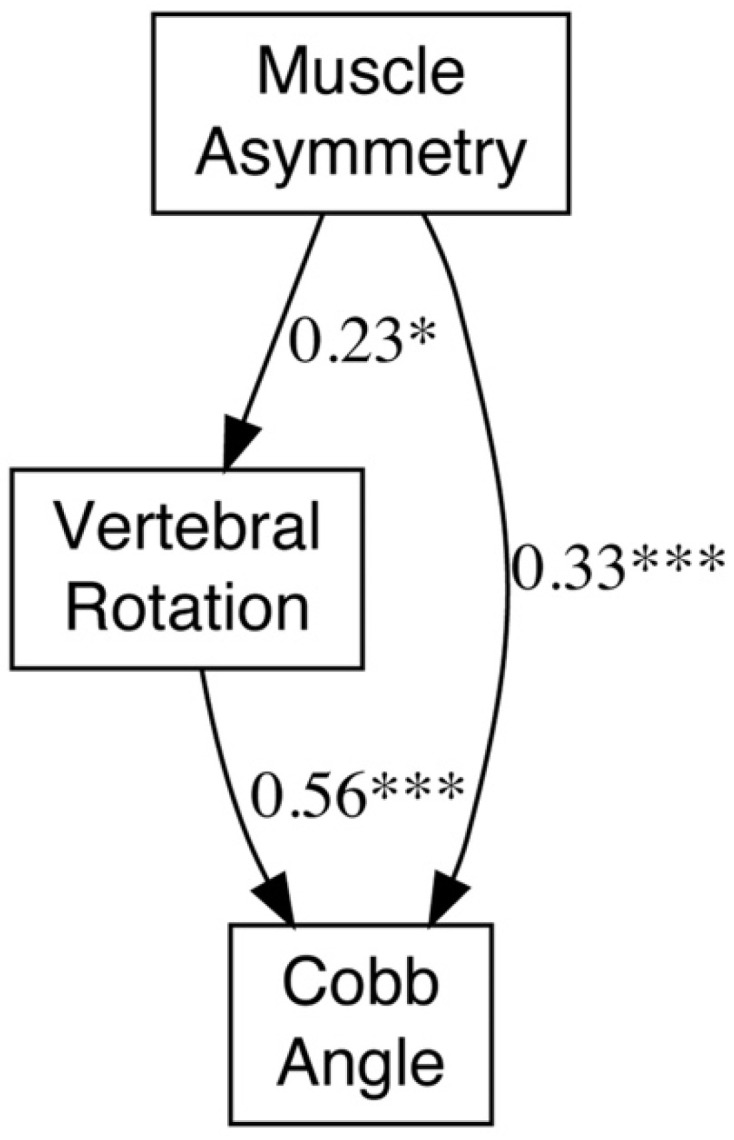
Structural Equation Modeling (SEM) of the relationships among muscle asymmetry, AVR, and Cobb angle. Path diagram illustrating the relationships among Muscle Asymmetry, AVR, and Cobb Angle. Numbers on arrows represent standardized path coefficients (β). Solid arrows indicate statistically significant pathways (*p* < 0.05). * Significance levels: *p* < 0.05 (*), *p* < 0.001 (***).

In an additional SEM analysis (Figure 6), muscle asymmetry had a standardized path coefficient of 0.232 to AVR (*p* = 0.032), AVR had a standardized path coefficient of 0.559 to Cobb angle (*p* < 0.001), and muscle asymmetry had a direct standardized path coefficient of 0.326 to Cobb angle (*p* = 0.001). The bootstrap indirect effect was 0.130 (95% CI 0.017 to 0.311; *p* = 0.044). These findings were interpreted as statistically compatible with the observed association pattern and not as evidence of temporal sequence.

## 4. Discussion

Previous imaging studies have repeatedly confirmed that structural alterations in the paraspinal muscles are prevalent among patients with AIS, with the asymmetric distribution of CT-derived low-attenuation change being the most common observation [18,19]. Particularly at the level of the apical vertebra of the major curve, the degree of muscle composition asymmetry on the concave side is consistently reported to be significantly more pronounced than that on the convex side across numerous CT and MRI studies [2,18,20]. However, prior research has been largely confined to morphological descriptions, and the precise role of this muscle asymmetry in the formation and progression of the three-dimensional spinal deformity remains unclear [6,21].

Building upon this foundation, the present study utilized quantitative CT analysis to further confirm the presence of significant asymmetry in paraspinal low-attenuation signal at the apical region in patients with AIS, with greater severity observed on the concave side. Notably, this asymmetry was absent at the stable vertebral level, which is removed from the center of the deformity, indicating distinct segmental specificity. Furthermore, SEM showed an association between the degree of paraspinal fatty infiltration asymmetry, AVR, and overall curve severity. These findings suggest that paraspinal muscle quality asymmetry coincides with the three-dimensional deformity of AIS and may reflect a localized imaging correlate of deformity severity rather than a uniformly distributed secondary change.

The increased concave-sided low-attenuation change at the apical region observed in this study is highly consistent with previous MRI findings. Studies by Duncombe et al. and Berry et al. have confirmed that the intramuscular fat content of the multifidus and longissimus muscles on the concave side is significantly higher than that on the convex side in patients with AIS, with this asymmetry being most pronounced at the apex [2,18]. A similar pattern of concave-sided muscle asymmetry is also observed in degenerative lumbar scoliosis (DLS) and is closely correlated with deformity severity and vertebral rotation [22,23,24]. This supports the possibility of a broader muscle-bone relationship in scoliosis, although the direction of causality remains unclear. Notably, while some previous studies have focused on asymmetry in muscle volume or CSA [1,17], morphological volume does not equate to contractile function. Histological evidence from Shahidi et al. indicates that severe fatty replacement and fibrosis can occur even in muscles without significant volumetric reduction [21]. Therefore, compared to changes in quantity, the focus of this study on a CT-derived composition surrogate provides a pragmatic imaging approximation of altered muscle quality, but it still requires cautious interpretation and should not be equated with histologically confirmed fatty infiltration or direct functional impairment.

The association between muscle asymmetry and AVR deserves particular caution. Pearson correlation and linear regression showed borderline results, whereas Spearman correlation indicated a modest association. In addition, AVR across vertebral levels showed only a trend toward higher values at the pedicle/apical level after repeated-measures correction. These findings suggest that the relationship between muscle asymmetry and rotation is weaker and more method-dependent than the relationship between muscle asymmetry and Cobb angle.

This asymmetric muscle composition pattern may reflect a combination of local adaptation, neuromotor asymmetry, and systemic biological influences [25]. Previous studies have demonstrated that patients with AIS exhibit widespread disturbances in bone metabolism and endocrine homeostasis, including melatonin, leptin, and oxidative stress pathways [26,27,28]. Molecular-level studies further suggest that the downregulation of estrogen receptor 1 (ESR1) expression in concave paraspinal muscle progenitor cells can impair myogenesis and promote adipogenesis [16]. Concurrently, morphological abnormalities and denervation changes at the neuromuscular junction (NMJ) on the convex side indicate the involvement of neurogenic factors [29]. The coexistence of concave composition changes and convex neurogenic remodeling collectively disrupts the dynamic mechanical balance on both sides of the spine [2,18,22]. The macroscopic low-attenuation change captured by CT imaging in this study is likely one imaging-level correlate of these micro-level pathological changes at the histological level, reflecting the complexity of muscle pathology in AIS.

The additional SEM analysis summarized the statistical relationships, but these results does not establish causality or temporal sequence. A cross-sectional SEM can be compatible with several competing biological explanations, including reverse adaptation or bidirectional feedback between muscle and deformity.

These findings have cautious clinical implications. CT-derived paraspinal muscle asymmetry may provide complementary information when characterizing three-dimensional deformity severity in severe AIS, but the present study does not suppoer its use as it should be used as a standalone prognostic or therapeutic marker. Longitudinal studies with MRI-based muscle characterization, functional testing, and repeatability data are needed to test whether these CT-derived surrogates are modifiable, prognostic, or treatment-responsive.

There are certain limitations to this study. First, although SEM was performed as an additional analysis, the cross-sectional design precludes causal inference. Future longitudinal follow-up studies are required to verify whether muscle pathology precedes the aggravation of skeletal deformity. Second, compared to MRI, CT has lower soft tissue contrast. Although we employed high-precision threshold segmentation algorithms, minor intramuscular fat deposits may still have been missed. The HU threshold used in this study should be understood as an operational attenuation-based definition rather than a tissue-specific gold standard. Finally, due to ethical considerations, this study used the stable vertebra as an internal control. While this method has been proven effective in previous studies [17], it precludes the assessment of whether patients with AIS exhibit systemic changes in the muscular system. Additional limitations include manual ROI tracing, potential slice angulation differences between the concave and convex sides, use of a universal rather than muscle-specific phantom, the fact that repeatability analysis was available for the apical muscle measurements but not for all historical imaging variables or all derived metrics, the severe-curve skew of the cohort, and the fact that averaging three axial slices still does not provide volumetric muscle assessment.

## 5. Conclusions

In conclusion, significant asymmetric CT-derived low-attenuation change exists in the apical region of patients with AIS. In this severe AIS cohort, paraspinal muscle asymmetry was consistently associated with Cobb angle and showed a weaker, more method-dependent association with AVR. In multivariable regression, both AVR and muscle asymmetry remained independently associated with Cobb angle, explaining approximately half of the variance in deformity severity. These findings show that paraspinal muscle asymmetry is associated with three-dimensional deformity severity, though the cross-sectional design precludes causal inference.

## Figures and Tables

**Table 1:** Demographic and baseline characteristics of the study population.

Characteristic	Mean ± SD	*n*
Demographics		
Age (years)	14.2 ± 3.1	68
Height (m)	1.47 ± 0.20	68
Weight (kg)	39.5 ± 11.2	68
BMI (kg/m^2^)	17.9 ± 2.7	68
Skeletal Parameters		
Main Curve Cobb Angle (degree)	76.6 ± 17.8	68
AVR (degree)	24.7 ± 8.1	68
Muscle Parameters		
Apical Muscle Asymmetry (%)	17.6 ± 15.5	68

**Table 2:** Comparison of paraspinal muscle characteristics between concave and convex sides.

Vertebral Level/Parameter	Concave (Mean ± SD)	Convex (Mean ± SD)	*p* Value
Apical Vertebra Fat Infiltration Rate (FIR, %)	33.4 ± 14.0	15.7 ± 6.8	<0.001
Stable Vertebra Fat Infiltration Rate (FIR, %)	33.0 ± 13.1	33.1 ± 13.1	0.551
Stable Vertebra Total Muscle Area (mm^2^)	2328.6 ± 1880.8	2319.6 ± 1867.1	0.168
Stable Vertebra Mean Signal Intensity	13,054.5 ± 13,853.5	13,313.0 ± 14,055.4	0.103

Note: The apical section focuses on the primary compositional endpoint (FIR), whereas the stable section additionally includes internal-control descriptors of area and signal intensity.

**Table 3:** Correlation analysis of muscle asymmetry, AVR, and Cobb angle.

Relationship	Result
Muscle asymmetry vs. AVR	Pearson r = 0.232, *p* = 0.0584; Spearman rho = 0.302, *p* = 0.0131
Muscle asymmetry vs. Cobb angle	Pearson r = 0.456, *p* = 0.0001; Spearman rho = 0.430, *p* = 0.0003
AVR vs. Cobb angle	Pearson r = 0.635, *p* < 0.001; Spearman rho = 0.641, *p* < 0.001

AVR: Vertebral axial rotation.

**Table 4:** Linear regression models relating muscle asymmetry, AVR, and Cobb angle.

Model	Predictor	β (95% CI)	*p* Value	R^2^
Model A	Muscle_Asymmetry	0.122 (−0.005 to 0.249)	0.0584	0.054
Model B	Muscle_Asymmetry	0.524 (0.271 to 0.778)	0.0001	0.208
Model C	Mean_AVR	1.222 (0.827 to 1.617)	<0.001	0.5039
Model C	Muscle_Asymmetry	0.375 (0.167 to 0.583)	0.0006	0.5039

## Data Availability

The datasets generated during and analyzed during the current study are not publicly available due to the protection of patient privacy but are available from the corresponding author on reasonable request.

## Supplementary Materials

The following supporting information can be downloaded at: https://www.mdpi.com/article/10.3390/jcm15083084/s1, Table S1: FIR summaries and paired side-to-side comparisons under the two HU threshold definitions; Table S2: Agreement of the patient-level muscle asymmetry index between HU threshold definitions; Table S3: Correlation analyses under the two HU threshold definitions; Table S4: Linear regression models under the two HU threshold definitions.
