# Supplementary material for: CT-Derived Paraspinal Muscle Asymmetry Is Associated with Deformity Severity in Adolescent Idiopathic Scoliosis: A Quantitative CT Study
Source: J Clin Med. 2026 Apr 17;15(8):3084. doi: 10.3390/jcm15083084 (PMC13117103; doi:10.3390/jcm15083084)
Supplement: Supplementary file 1 [file jcm-15-03084-s001.zip › jcm-4169991-supplementary.pdf]

Table S1. FIR summaries and paired side-to-side comparisons under the two HU threshold definitions

Region	Threshold definition	n	Mean concave FIR (%)	Mean convex FIR (%)	Mean difference (%)	Concave SD (%)	Convex SD (%)	P value
Apex	Primary (-110 to -35 HU)	30	24.41	23.57	0.84	9.06	6.29	0.6071
Apex	Alternative (-120 to -30 HU)	30	19.67	19.11	0.56	9.40	7.14	0.9018
Stable vertebra	Primary (-110 to -35 HU)	30	18.51	17.76	0.75	5.52	5.38	0.8531
Stable vertebra	Alternative (-120 to -30 HU)	30	14.35	14.07	0.28	5.80	5.61	0.8050

Table S2. Agreement of the patient-level muscle asymmetry index between HU threshold definitions

Region	n	Pearson r	P value	Mean absolute difference (%)	Maximum absolute difference (%)
Apex	30	0.959	<0.001	2.16	5.81
Stable vertebra	30	0.965	<0.001	1.75	3.90

Table S3. Correlation analyses under the two HU threshold definitions

Region	Threshold definition	Relationship	Pearson r	Pearson P value	Spearman rho	Spearman P value
Apex	Primary (-110 to -35 HU)	Muscle asymmetry vs Mean AVR	-0.058	0.7619	0.019	0.9210
Apex	Primary (-110 to -35 HU)	Muscle asymmetry vs Mean Cobb	-0.063	0.7417	0.007	0.9711

Region	Threshold definition	Relationship	Pearson r	Pearson P value	Spearman rho	Spearman P value
Apex	Alternative (-120 to -30 HU)	Muscle asymmetry vs Mean AVR	0.004	0.9821	0.034	0.8564
Apex	Alternative (-120 to -30 HU)	Muscle asymmetry vs Mean Cobb	-0.042	0.8270	-0.019	0.9210
Stable vertebra	Primary (-110 to -35 HU)	Muscle asymmetry vs Mean AVR	0.208	0.2702	0.203	0.2817
Stable vertebra	Primary (-110 to -35 HU)	Muscle asymmetry vs Mean Cobb	0.327	0.0781	0.323	0.0819
Stable vertebra	Alternative (-120 to -30 HU)	Muscle asymmetry vs Mean AVR	0.087	0.6464	-0.025	0.8969
Stable vertebra	Alternative (-120 to -30 HU)	Muscle asymmetry vs Mean Cobb	0.288	0.1229	0.176	0.3523

Table S4. Linear regression models under the two HU threshold definitions

Region	Threshold definition	Model	Term	Estimate (95% CI)	P value	R2
Apex	Primary (-110 to -35 HU)	Mean_AVR ~ Muscle_Asymmetry	Intercept	23.409 (20.336 to 26.482)	<0.001	0.0033
Apex	Primary (-110 to -35 HU)	Mean_AVR ~ Muscle_Asymmetry	Muscle_Asymmetry	-0.053 (-0.410 to 0.303)	0.7619	0.0033
Apex	Primary (-110 to -35 HU)	Mean_Cobb ~ Muscle_Asymmetry	Intercept	76.718 (70.306 to 83.131)	<0.001	0.0039
Apex	Primary (-110 to -35 HU)	Mean_Cobb ~ Muscle_Asymmetry	Muscle_Asymmetry	-0.121 (-0.865 to 0.623)	0.7417	0.0039
Apex	Primary (-110 to -35 HU)	Mean_Cobb ~ Mean_AVR +	Intercept	37.768 (25.476 to 50.060)	<0.001	0.6372

Region	Threshold definition	Model	Term	Estimate (95% CI)	P value	R2
		Muscle_Asymmetry				
Apex	Primary (-110 to -35 HU)	Mean_Cobb ~ Mean_AVR + Muscle_Asymmetry	Mean_AVR	1.664 (1.167 to 2.161)	<0.001	0.6372
Apex	Primary (-110 to -35 HU)	Mean_Cobb ~ Mean_AVR + Muscle_Asymmetry	Muscle_Asymmetry	-0.032 (-0.491 to 0.426)	0.8862	0.6372
Apex	Alternative (-120 to -30 HU)	Mean_AVR ~ Muscle_Asymmetry	Intercept	23.362 (20.294 to 26.431)	<0.001	0.0000
Apex	Alternative (-120 to -30 HU)	Mean_AVR ~ Muscle_Asymmetry	Muscle_Asymmetry	0.004 (-0.327 to 0.335)	0.9821	0.0000
Apex	Alternative (-120 to -30 HU)	Mean_Cobb ~ Muscle_Asymmetry	Intercept	76.658 (70.258 to 83.058)	<0.001	0.0017
Apex	Alternative (-120 to -30 HU)	Mean_Cobb ~ Muscle_Asymmetry	Muscle_Asymmetry	-0.074 (-0.765 to 0.616)	0.8270	0.0017
Apex	Alternative (-120 to -30 HU)	Mean_Cobb ~ Mean_AVR + Muscle_Asymmetry	Intercept	37.729 (25.511 to 49.948)	<0.001	0.6390
Apex	Alternative (-120 to -30 HU)	Mean_Cobb ~ Mean_AVR + Muscle_Asymmetry	Mean_AVR	1.666 (1.171 to 2.162)	<0.001	0.6390
Apex	Alternative (-120 to -30 HU)	Mean_Cobb ~ Mean_AVR + Muscle_Asymmetry	Muscle_Asymmetry	-0.080 (-0.504 to 0.343)	0.6998	0.6390
Stable vertebra	Primary (-110 to -35 HU)	Mean_AVR ~ Muscle_Asymmetry	Intercept	23.203 (20.192 to 26.213)	<0.001	0.0432

Region	Threshold definition	Model	Term	Estimate (95% CI)	P value	R2
Stable vertebra	Primary (-110 to -35 HU)	Mean_AVR ~ Muscle_Asymmetry	Muscle_Asymmetry	0.215 (-0.176 to 0.605)	0.2702	0.0432
Stable vertebra	Primary (-110 to -35 HU)	Mean_Cobb ~ Muscle_Asymmetry	Intercept	76.086 (70.014 to 82.159)	<0.001	0.1067
Stable vertebra	Primary (-110 to -35 HU)	Mean_Cobb ~ Muscle_Asymmetry	Muscle_Asymmetry	0.704 (-0.085 to 1.492)	0.0781	0.1067
Stable vertebra	Primary (-110 to -35 HU)	Mean_Cobb ~ Mean_AVR + Muscle_Asymmetry	Intercept	39.124 (27.170 to 51.079)	<0.001	0.6639
Stable vertebra	Primary (-110 to -35 HU)	Mean_Cobb ~ Mean_AVR + Muscle_Asymmetry	Mean_AVR	1.593 (1.104 to 2.082)	<0.001	0.6639
Stable vertebra	Primary (-110 to -35 HU)	Mean_Cobb ~ Mean_AVR + Muscle_Asymmetry	Muscle_Asymmetry	0.362 (-0.142 to 0.866)	0.1525	0.6639
Stable vertebra	Alternative (-120 to -30 HU)	Mean_AVR ~ Muscle_Asymmetry	Intercept	23.341 (20.287 to 26.394)	<0.001	0.0076
Stable vertebra	Alternative (-120 to -30 HU)	Mean_AVR ~ Muscle_Asymmetry	Muscle_Asymmetry	0.085 (-0.292 to 0.463)	0.6464	0.0076
Stable vertebra	Alternative (-120 to -30 HU)	Mean_Cobb ~ Muscle_Asymmetry	Intercept	76.453 (70.325 to 82.580)	<0.001	0.0829
Stable vertebra	Alternative (-120 to -30 HU)	Mean_Cobb ~ Muscle_Asymmetry	Muscle_Asymmetry	0.588 (-0.169 to 1.346)	0.1229	0.0829
Stable vertebra	Alternative (-120 to -30 HU)	Mean_Cobb ~ Mean_AVR + Muscle_Asymmetry	Intercept	38.505 (27.063 to 49.947)	<0.001	0.6849

Region	Threshold definition	Model	Term	Estimate (95% CI)	P value	R2
Stable vertebra	Alternative (-120 to -30 HU)	Mean_Cobb ~ Mean_AVR + Muscle_Asymmetry	Mean_AVR	1.626 (1.161 to 2.090)	<0.001	0.6849
Stable vertebra	Alternative (-120 to -30 HU)	Mean_Cobb ~ Mean_AVR + Muscle_Asymmetry	Muscle_Asymmetry	0.449 (-0.005 to 0.904)	0.0526	0.6849
